# Addenbrooke's Hospital, Cambridge

**Published:** 1904-11-12

**Authors:** 


### HOSPITAL ADMINISTRATION.

### CONSTRUCTION AND ECONOMICS.

### ADDENBROOKE'S HOSPITAL, CAMBRIDGE.

RESIGNATION OF THE SUPERINTENDENT, THE MATRON, AND THE SECRETARY.

The management of Addenbrooke's Hospital, Cambridge, recently reflects little credit upon the Governors who have been charged with the duty of administering its affairs. Since 1898, when Sir Henry Burdett was called in to advise the Governors what steps should be taken with a view to improving the system and the management, great changes have been effected in the constitution and also in the methods of procedure. The open board has been abolished and a committee of management, consisting of 24 Governors, has been set up; an improved system of electing the honorary medical staff has been adopted; the condition of the nursing and the position of the probationers especially have been improved; the system of accounts, which was formerly unsatisfactory, has now been modelled upon the uniform system, whereby every one interested in the hospital can easily ascertain how the money is spent and what is the relative cost of each department of the institution; an executive committee and a finance committee are now charged with the immediate control of the business of the hospital. All these changes have greatly improved on paper the system of administration. In practice, however, we are informed that the management of Addenbrooke's Hospital at the present time is not satisfactory, owing to the want of an annealing influence which will prevent personal differences and prejudices from interfering with the conduct of the business of the committee of management and the other committees. These differences, which have frequently been rife at this hospital, are in fact the real cause of the unsatisfactory condition of its administration to-day.

It is not easy for men of business to realise the pettiness which for the most part has been the bane of this important hospital. Members of a hospital committee who are qualified to occupy that responsible position must rise above petty details and set their faces against wasting time upon the purchase of mouse-traps and every other small article, which in the ordinary course it is necessary to supply for the purposes of the hospital. It is not the duty of a committee or its members to mix themselves up with every petty matter, but to grasp the reins of authority with a firm hand, and to see that the general administration in all departments is efficient and good. It is no use keeping a dog and barking oneself. A hospital committee should trust its officers and leave them to carry out the duties entrusted to them without unnecessary meddling. These truths have apparently yet to be learnt by several members of the committee of Addenbrooke's Hospital; but, as we understand and can well believe that they form but a minority of the present governing body, we venture to hope that the election of Mr. A. J. Pell as chairman and Mr. T. Musgrave Francis as deputy chairman will result in ending the present *impasse* by introducing business methods into the proceedings of all the committees responsible for the affairs of this institution. Mr. Pell is a member of the County Council; he has had considerable experience in public business and the management of committees, and we hope he will exert his authority during his term of office to prevent waste of time, to avoid the exhibition of personal differences amongst members of the committee, and generally to purge the management of the personalities and pettiness which are stated to have so long crippled the efficient working of the various committees at Cambridge.

At the present time we have reason to believe that there is no proper system of ordering goods and supplies of all kinds at Addenbrooke's Hospital. A committee of ladies has shown, as the accounts themselves exhibit, that a considerable saving might be effected in the expenditure on provisions especially. If it be true, as we understand to be the case, that instead of a system whereby no article is allowed to enter the hospital except upon a written order signed by a responsible official, the practice has been for various people to order things on the telephone and then for them to be received at the hospital without an invoice, we can quite understand how it is that although many words have been expended upon criticism, nothing effectual in the way of the control of expenditure has yet been attained. Whatever may be the causes which have led to the present deadlock, which has landed this hospital in the position of losing at the same time its superintendent, its secretary, and its matron, it is hoped that Mr. Pell will devote his personal attention, as chairman, to the crisis which has thus arisen. If so, steps should be taken to so reorganise the staff as to secure that whoever may be appointed to fill the vacant posts will have assigned to them definite duties, and that within the limitations of these duties every officer will have fair treatment, a free hand, and the loyal support of the committee which may make the new appointments. It is not often that the chairman of a hospital has so good an opportunity, as Mr. Pell now has, of helping the community amongst which he resides. We hope he may mark his term of office by the introduction of business methods, not only within the hospital but within the board-room, where their absence in the past has led to an inordinate waste of time, and to a breakdown so marked, as to be little creditable to anyone who may be responsible for the ineptitudes which have led to the present *impasse* at Addenbrooke's Hospital, Cambridge.

